# History of a Case of Traumatic Tetanus, Successfully Treated

**Published:** 1821-12

**Authors:** Augustin Mercier, Joseph Parant

**Affiliations:** Member of the Royal College of Surgeons in London, &c.; Member of the Royal College of Surgeons in London, &c.


*History of a Case of Traumatic Tetanus, successfully treated.* By Messrs. AUGUSTIN MERCIER and JOSEPH PARANT, Members of the Royal College of Surgeons in London, &c.

MR. FRANCIS MAILHIOT, fifty years of age, of a sanguineous temperament, living in the upper town of Quebec, and enjoying a good state of health, received a wound on the 6th of June, 1821, while walking on the plains of Abraham, from a large nail penetrating the sole of his left foot. The pain which ensued was so great as to oblige him to return home in a coach. The wound healed over in a few days, the cicatrix remaining indurated. The patient resumed his usual occupations until the 16th, when, on returning from attending the funeral of a friend, he felt great uneasiness in the extremity originally affected, so that, by the time he reached his own house, he was suffering from intense pain, which induced him to apply a small quantity of tar over the indurated surface. Finding, however, that the pain increased every moment, and being greatly agitated, he desired our attendance, at about 7 P.M. We found Mr. Mailhiot labouring under an incipient opisthotonos: he was complaining of great pain in the affected foot, leg, and thigh; the muscles of the trunk appeared greatly contracted; he had an involuntary grinding of the teeth; and was constantly endeavouring, with a handkerchief thrown behind his head, to impede the contraction of the muscles of the back, by which his head was drawn backwards. His pulse was hard, and beat 120 in a minute.

After a due consideration of the case, we resolved to re-open the wound, and to immerse the foot in warm water, with a view to encourage the bleeding. This gave the patient some minutes' relief; but, the paroxysms returning soon after with increased violence, he was bled, in a sitting posture, *ad deliquium animi*. This bleeding gave him much ease, by relaxing, for a short time, the muscular system. We afterwards introduced into the wound a piece of lint dipped in spirits, in a burning state, by which operation he was thrown into a violent paroxysm, which lasted but a few minutes, and after which he felt much relieved. We gave him gr. xx. calomel. At 9 P.M. he felt easier: the calomel had not operated; the pulse was yet hard and frequent; the spasmodic contraction of the muscles had diminished. He was again bled to twelve ounces. The same quantity of calomel, with the addition of one ounce of Epsom salts, was prescribed.

On the 17th, at 6 A.M. felt easier; had passed a good night; pulse 100; no alvine evacuation. We gave him one ounce of Epsom salts, and ordered him to be put into a warm bath.—At 9 A.M. no alvine evacuation; pulse 100. V. S. ℥xx. after which the pulse fell to 80. Rep. magn. sulphatis, ℥j.—At 12 P.M. the bowels had been well opened; the blood drawn in the morning had the inflammatory appearance in a slight degree. We ordered him to be put into a warm bath.—At 2 P.M. he felt much refreshed by the warm bath; pulse 82. He took ℥j. ol. ricini.—At 7 P.M. had slight paroxysms; pulse softer and 80.—At 9 P.M. pulse had risen to 100, was hard and vibrating: the patient had experienced a return of the paroxysms. V.S. ad ℥xvj.; the warm bath, and ol. ricini ℥j.

On the 18th, at 7 A.M. the pulse quick, hard, and intermittent, 110; has had four alvine evacuations since yesterday evening, and passed a very restless night. He complains of great pain at the pit of the stomach, and has continual inclinations to vomit. V.S. ad ℥viij. which reduced the pulse to 80, and relieved the pain at the stomach. The wound was examined, and found in a state of suppuration; its edges were less hard. The mistura antimonialis was had recourse to.—At 12 P.M. the pulse 100. Gave him one ounce of Epsom salts to take immediately; and, an hour after, a pill composed of elaterium, gamboge, and aloes, p. gr. ij.—At 3 P.M. no alvine evacuation. The same pill to be repeated.—At 6 P.M. has had an evacuation, and feels a great deal easier.—At 8 P.M. has had an evacuation; pulse 90; skin moist; feels no pain except in the foot.

On the 19th, 7½ A.M. pulse hard and intermittent, 100; the bowels had not been relieved, and he had passed a very restless night; abdomen much distended. Rep. pilula ut heri et magnesiæ sulphatis, ℥j.—At 12 P.M. has had two evacuations; pulse 96; the belly continues very tense; complains of severe pain in the head, as well as in the wound and along the affected nerve. Ordered him to be put into a warm bath.—At 4½ P.M. has had a copious evacuation; belly natural. As the wound appeared to be healing up, lint, dipped in spirits and in a burning state, was again introduced into it.—At 7 P.M. felt entirely refreshed and easy.

20th, 8 A.M. abdomen natural; no pain in any part of the body. Ordered a little castor oil. Pulse natural. The patient appears to be in a convalescent state; and has since completely recovered.

We need not remark, that the antiphlogistic regimen was strictly attended to throughout the duration of the disease.

It may be stated, as the opinion of the attending surgeons, that copious bleeding and the cauterizing of the wound have done, in this instance, more good than any other part of the medical treatment.

*Quebec; 18th August, 1821.*

